# 

**Published:** 1875-11

**Authors:** 


### CONTENTS:

### ORIGINAL COMMUNICATIONS.

ARTICLE I.—Alternation a Law of Vital Action. By Dr. L. C. Ingersoll.......... 289

ARTICLE II.—Dental Education. By Dr. S. J. Cobb.............................. 303

### SELECTED ARTICLES.

ARTICLE III.—The American Dental Association.................................. 311
ARTICLE IV.—Exposed Pulps. By Wm. C. Wardlaw, D. D. S.......................... 324
ARTICLE V.—Tumors of the Tongue. By M. Molliere................................. 327

### EDITORIAL, ETC.

Salicylic Acid as an Antiseptic, etc.............................................. 330
Susquehanna Dental Association..................................................... 333

### MONTHLY SUMMARY.

Adulteration of Beeswax............................................................ 334

A Photographic Wonder............................................................... 335

External Use of Chloral Hydrate..................................................... 336

The Anti-Neuralgic Properties of Essence of Mint.................................... 336

